# Global determinants of home range sizes in felids: Evidence of human disturbance impact

**DOI:** 10.1111/1365-2656.70227

**Published:** 2026-02-04

**Authors:** Arthemis Moraru, Stefano Anile, Sébastien Devillard

**Affiliations:** ^1^ Université Claude Bernard Lyon 1, LBBE, UMR 5558, CNRS, VAS Villeurbanne France; ^2^ Université Claude Bernard Lyon 1, LEHNA UMR 5023, CNRS, ENTPE Villeurbanne France; ^3^ Department of Biological, Chemical and Pharmaceutical Sciences and Technologies (STEBICEF)—Section of Botany, Anthropology, Zoology University of Palermo Palermo Italy; ^4^ National Biodiversity Future Center (NBFC) Palermo Italy; ^5^ Department of Research and Conservation Wildlife Initiative Italy Verona Italy

**Keywords:** drivers, felid, felid richness, global, home range, human disturbance, meta‐analysis, space use

## Abstract

1. Home range (HR) is a key indicator of animal spatial ecology. HR size, shape, location and habitat composition reflect both species' ecological requirements and their responses to anthropogenic stressors. *Felidae*, a charismatic taxon, faces escalating threats mainly due to habitat degradation and human–wildlife conflict. Understanding the ecological and anthropogenic drivers of HR size variation is therefore critical for their conservation.
2. To address this gap and explore these factors at a global scale for the entire taxon, we used the *HomeRange* database—a global database with HR values across 960 different mammal species—complemented with about 20% additional records, to compile 1137 individual HR size estimates from 29 out of 40 recognized wild felid species. We applied generalized linear mixed models to assess the influence of intrinsic, methodological, ecological and anthropogenic factors on space use.
3. HR size was shaped by multiple drivers. It increased with body mass (0.94 ± 0.16; *p* < 10^−8^) and was larger in males than in females (0.51 ± 0.07; *p* < 10^−13^), consistent with higher energy demands and sex‐specific reproductive strategies. HR size decreased with increasing productivity (−0.37 ± 0.07; *p* < 10^−7^) and felid richness (−0.24 ± 0.10; *p* = 0.02), suggesting reduced spatial requirements in resource‐rich areas and under interspecific competition. HR size also decreased with increasing croplands (HR: −0.50 ± 0.14; *p* < 10^−3^) and pastures (HR: −0.16 ± 0.07; *p* = 0.02)—both human footprint proxies—which may reflect multiple causes such as anthropogenic food sources, habitat loss or movement restriction from infrastructures associated with agriculture.
4. Our results reinforce the role of well‐known established HR size's predictors such as body mass, sex and primary productivity while highlighting the impact of less frequently investigated factors (i.e. felid richness and agricultural land‐use). Our findings emphasize the importance of incorporating a broad range of biological, environmental and methodological predictors when studying space use across a taxonomic group. Our approach provides novel insights into habitat requirements and the effects of anthropogenic pressures, which can ultimately lead to improved conservation strategies for felids.

## Graphical abstract

This study is the first to demonstrate the influence of anthropogenic factors and species richness in felids on home range size across the entire felid taxon. Felids are a taxon of major interest in conservation biology, and gaining a comprehensive view of their spatial ecology will help derive global conservation recommendations.

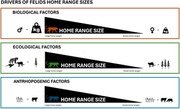

## Résumé

1. Le domaine vital (home range, HR) est un indicateur clé de l'écologie spatiale des animaux. Sa taille, sa forme, sa localisation et la composition de l'habitat reflètent à la fois les exigences écologiques des espèces et leurs réponses aux pressions anthropiques. Les félidés, un taxon charismatique, font face à des menaces croissantes, principalement dues à la dégradation des habitats et aux conflits avec les humains. Comprendre les facteurs écologiques et anthropiques expliquant la variation de la taille du domaine vital est donc crucial pour leur conservation.
2. Afin d'explorer ces facteurs à l'échelle globale pour l'ensemble du taxon, nous avons utilisé la base de données HomeRange — une base mondiale regroupant des valeurs de HR pour 960 espèces de mammifères — complétée par environ 20% de données supplémentaires. Nous avons ainsi compilé 1 137 estimations individuelles de taille de HR provenant de 29 des 40 espèces de félidés sauvages reconnues. Des modèles linéaires généralisés mixtes ont été appliqués afin d'évaluer l'influence de facteurs intrinsèques, méthodologiques, écologiques et anthropiques sur l'utilisation de l'espace.
3. La taille du HR était influencée par de multiples facteurs. Elle augmentait avec la masse corporelle (0,94 ± 0,16; *p* < 10^‐8^) et était plus élevée chez les mâles que chez les femelles (0,51 ± 0,07; *p* < 10^‐13^), en accord avec des besoins énergétiques plus importants et des stratégies reproductives spécifique au sexe. La taille du HR diminuait avec l'augmentation de la productivité primaire (‐0,37 ± 0,07; *p* < 10^‐7^) et de la richesse spécifique en félidés (‐0,24 ± 0,10; *p* = 0,02), suggérant des besoins spatiaux réduits dans les zones riches en ressources et sous l'effet de la compétition interspécifique. La taille du HR diminuait également avec l'augmentation des surfaces cultivées (‐0,50 ± 0,14; *p* < 10^‐3^) et des pâturages (‐0,16 ± 0,07; *p* = 0,02), deux indicateurs de l'empreinte humaine, ce qui pourrait refléter différentes causes telles que la présence de ressources alimentaires d'origine anthropique, la perte d'habitat ou des restrictions de déplacement liées aux infrastructures agricoles.
4. Nos résultats confirment le rôle de prédicteurs bien établis de la taille du HR, tels que la masse corporelle, le sexe et la productivité primaire, tout en mettant en évidence l'impact de facteurs moins fréquemment étudiés, comme la richesse spécifique en félidés et l'usage agricole des terres. Ils soulignent l'importance d'intégrer un large éventail de prédicteurs biologiques, environnementaux et méthodologiques lors de l'étude de l'utilisation de l'espace à l'échelle d'un groupe taxonomique. Notre approche apporte de nouvelles connaissances sur les exigences en matière d'habitat et les effets des pressions anthropiques, pouvant à terme conduire à l'amélioration des stratégies de conservation des félidés.

## 1 INTRODUCTION

Human activities are a driving force of contemporary global environmental change, resulting in dramatic declines in biodiversity (Butchart et al., 2010), degradation of ecosystems' stability (Hautier et al., 2015) and acceleration in species extinction rates (Barnosky et al., 2011). These changes lead to the ongoing sixth extinction crisis (Cowie et al., 2022), primarily driven by habitat destruction, fragmentation, overexploitation, pollution, invasive species and climate change. According to the IUCN (2024), 27% of mammals are threatened with extinction, with similar trends observed across other taxa (Cowie et al., 2022). Nearly half of Earth's land is transformed into agricultural or urban landscapes, with much of the remaining natural habitats fragmented by roads (Barnosky et al., 2012). This unprecedented rate of human disturbance can disrupt spatial ecology (Tucker et al., 2018), that is the way species interact with their habitat across space and time.

Contraction of suitable habitat can prevent individuals from meeting their resource requirements, leading to decreased fitness and usually declining wildlife populations (except when humans provide supplemental food; Šálek et al., 2015; Tucker et al., 2021), thereby increasing the risk of extinction (Zanin et al., 2021). Therefore, it is essential to determine the threshold at which available habitat becomes insufficient for an animal's survival, to identify species at risk and to understand the drivers of their decline, ultimately guiding wildlife conservation efforts to better protect threatened species, such as felids (Dickman et al., 2015).

*Felidae* (*Carnivora*) is a diverse taxon currently comprising 40 wild species distributed across the globe and organized into 14 genera (Kitchener et al., 2017). Felids currently face escalating threats due to habitat degradation and increasing human–wildlife conflict (IUCN, 2024; Ripple et al., 2014). Indeed, 18 felid species are listed as endangered or vulnerable according to the IUCN (2024) report. Furthermore, declines in felid populations may trigger cascading effects on ecosystem functioning, as these predators play a key role in regulating prey communities (Prugh et al., 2009). Despite their conservation appeal, driven by charismatic species such as lions (*Panthera leo*), tigers (*Panthera tigris*) and cheetahs (*Acinonyx jubatus*), many smaller felids remain poorly understood (Albert et al., 2018; Brodie, 2009). Moreover, to our knowledge, a comprehensive overview of spatial ecology across the Felidae family has yet to be carried out.

Home range (HR) is defined as the area an animal regularly and actively uses for essential activities such as feeding, mating and caring for offspring (Burt, 1943). HR hence reflects a trade‐off between the benefits and costs of space use shaped by the interaction between an individual and its environment. Previous studies have identified body mass, sex and age as important intrinsic factors that can shape an individual's space use by determining home range size in relation to resource requirements and reproductive success (Duncan et al., 2015). Besides individual and species traits, environmental drivers, some linked to human disturbance, have also been shown to influence HR size. For example, it has been shown that HR size decreases with ecosystem productivity and human disturbance. However, those studies focused on broader groups such as terrestrial mammals (Broekman et al., 2024) and carnivores (Hirt et al., 2021; Šálek et al., 2015), or at smaller scales, at the intraspecific or populational level, mostly for larger felid species such as jaguars (*Panthera onca*) (e.g. Thompson et al., 2021) and leopards (*Panthera pardus*) (Rodríguez Recio et al., 2022; Snider et al., 2021).

Yet, a comprehensive meta‐analysis synthesizing how environmental factors, both ecological and anthropogenic, influence HR size variation in felids at a global scale has been lacking. The *Felidae* family is an excellent animal model from both eco‐evolutionary and applied conservation perspectives because, despite their wide range of body sizes and habitats, felids share many ecological and behavioural traits. They are predominantly nocturnal, solitary, territorial and occupy similar trophic roles as predators. Pooling data across species allows us to identify general patterns in space use that would be difficult to detect within individual species as a result of limited sample sizes. Analysing the family—as a whole—can help highlight how shared traits and constraints shape HR size across related species, while also accounting for intraspecific and interspecific variation. From a conservation standpoint, most felids act as umbrella species: protecting their habitats can confer benefits to multiple sympatric species and ecosystems in general.

Understanding the processes underlying species' spatial ecology and the mechanisms that allow animals to cope with environmental change is essential for assessing their habitat requirements and developing evidence‐based conservation measures (Sutherland et al., 2004) that support their long‐term survival (Pe'er et al., 2014). Such measures are crucial for preserving wildlife in increasingly human‐modified landscapes and the more general they are, that is relevant to an entire taxon, the easiest is to deploy them at a large scale over the globe.

This study uses individual HR estimates for a broad range of felid species from published literature worldwide. In addition to intrinsic factors such as body mass and sex, we extracted extrinsic factors—environmental and anthropogenic—to capture variation across study sites. We hence examined how species‐specific traits, as well as ecological and anthropogenic factors, drive HR size variation in felids using a generalized linear mixed modelling (GLMM) framework to investigate the following hypotheses (see hypotheses in Table 1).

**TABLE 1:** Expected effects of intrinsic and environmental variables and their interactions on home range (HR) size and their biological significance.

Variable	Expected relationship	
Adult body mass	A larger body mass increases metabolic demands, requiring a larger HR size to fulfil them (Tucker et al., 2014)	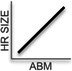
Sex	Males occupy a larger HR than females, as their territories overlap with multiple female home ranges, facilitating mating opportunities and resource control (Rodríguez Recio et al., 2022)	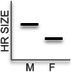
Elevation	Higher elevation areas are less impacted by humans and less productive thus leading to a larger HR size (Walton et al., 2017)	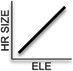
Net primary productivity	Higher productivity leads to higher prey availability and thus reduces HR size (Thompson et al., 2021)	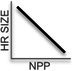
Felid richness	*Competition‐driven HR expansion (1)*: Higher felid richness increases interspecific interactions, expanding their movements to avoid competitors resulting in larger HRs (Oliveira et al., 2010) *Competition‐driven HR contraction (2)*: Higher felid richness increases interspecific interactions, leading to intensified niche differentiation and special efficiency, resulting in smaller HRs (Steinmetz et al., 2021) *Productivity‐driven HR contraction (2)*: Higher felid richness areas may be productive ‘hotspots’ with high prey availability allowing individuals to meet energetic needs within smaller areas, independent of competition (Chiaverini et al., 2025)	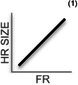 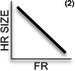
Human footprint index	Increased human pressures lead to a decrease in HR size by reducing available habitat (residential areas, agriculture), increasing fragmentation (roads) and creating a landscape of fear and human–wildlife conflicts (Hirt et al., 2021; Snider et al., 2021)	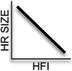
Human footprint index:body mass	Increased human pressures lead to a stronger decrease in HR size in larger felids with larger roaming areas (solid line) as they tend to encounter more humans and are more susceptible to human disturbances than small felids (dotted line) (Hirt et al., 2021)	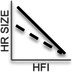
Human population density	Increased human population leads to a decrease in HR size by reducing available habitat (residential areas, agriculture, recreational activities …) and creating a landscape of fear and human–wildlife conflicts (Hirt et al., 2021; Snider et al., 2021)	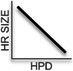
Human population density:body mass	Increased human population density leads to a stronger decrease in HR size in larger felids with larger roaming areas (solid line) as they tend to encounter more humans and are more susceptible to human disturbances than small felids (dotted line) (Hirt et al., 2021). They could also be more vulnerable to poaching or persecution	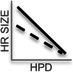
Road density	Higher road density increases habitat fragmentation which restricts movement, increases mortality risk, creates physical boundaries (Riley et al., 2006) and results in a smaller HR size	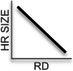
Road density:body mass	Larger felids (solid line) require extensive movement and thus are more affected by roads, experiencing stronger HR restrictions than smaller felids (dotted line) (Hirt et al., 2021)	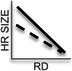
Croplands	Croplands fragment natural habitats and are often associated with higher road density and human presence, thus higher croplands density restricts movement and reduces HR size (Boron et al., 2020)	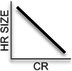
Croplands:body mass	Larger felids (solid line), which require extensive movement, are more affected by habitat fragmentation caused by croplands, resulting in stronger HR restrictions than smaller felids (dotted line). They could also benefit from more prey density in pastures, reducing their need to cover larger areas for food (Hirt et al., 2021)	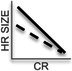
Pastures	Pastures are often associated with fences and human presence, thus restricting movement and reducing HR size (Cozzi et al., 2013; Jakes et al., 2018)	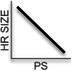
Pastures:body mass	Larger felids (solid line), which require extensive movement, are more affected by movement restrictions caused by pastures, resulting in stronger HR restrictions than smaller felids (dotted line). They could also benefit from more prey density in pastures, reducing their need to cover larger areas for food (Hirt et al., 2021)	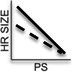

According to the body size hypothesis, we predicted that larger species would occupy a larger HR than smaller ones due to higher metabolic needs (Mcnab, 1963), keeping productivity constant. We further expect males to have a larger HR size than females because they have different needs in terms of reproductive strategies. According to the typically solitary social structure observed in all felid species except lions (Packer, 1987), males' HR often contain the territories of several females to have better access to reproduction (Erofeeva & Naidenko, 2012; Rodríguez Recio et al., 2022), while females' HR sizes are primarily driven by resource availability and offspring care (Klevtcova et al., 2021).

Moreover, we expected that felids exploit a smaller HR in highly productive landscapes where prey is abundant; this aligns with the habitat productivity hypothesis (Nilsen et al., 2005), which proposes that a rich environment allows animals to meet their metabolic needs over a smaller area. Furthermore, felid species often co‐occur in the same landscapes and adjust their behaviour—through spatial or temporal shifts—to reduce interspecific competition (Macdonald et al., 2010). In this context, felid richness (i.e. the number of cat species present in an area) likely reflects the intensity of this competition and could thus influence HR size. For example, both exploitative (e.g. competition for prey) and interference competition (e.g. intraguild predation) can drive spatial or temporal avoidance (Macdonald et al., 2010). Sympatric species can hence respond to intraspecific competition by adjusting their space use—either expanding or contracting their HRs depending on the dominant mechanism at play. Smaller felids are often displaced to suboptimal habitats (Monterroso et al., 2020; Vanak et al., 2013) and may compensate by expanding their HRs to access sufficient resources or minimize risky encounters (Cristescu et al., 2013; Oliveira et al., 2010). Under this scenario, higher felid richness increases competitive pressure, resulting in HR expansion as species attempt to avoid each other (competition‐driven HR expansion hypothesis). However, competition can also promote coexistence through niche differentiation and more efficient space use (Steinmetz et al., 2021). In carnivore communities, larger, dominant species tend to broaden their diets, whereas smaller, subordinate competitors become more specialized (Vogel et al., 2019). Moreover, HR size is linked to niche breadth, with the direction of the relationship depending on body size: large specialists tend to have larger HRs than large generalists, while small generalists have larger HRs than small specialists (Huang et al., 2021). Together, these studies suggest that competition‐driven niche differentiation could lead to an overall reduction in HR size with increasing felid richness: dominant species may adopt broader diets (niche expansion), while more subordinate species become more specialized (niche compression), both optimizing foraging strategies and reducing HR size (competition‐driven HR contraction hypothesis). Overall, competition among felid species is likely dictated by body mass—being strongest between species of similar size—and is further modulated by spatial and temporal niche differentiation, as well as prey‐preference factors. Alternatively, felid‐rich environments may simply reflect biodiversity hotspots, capable of sustaining multiple predator species and abundant and diverse prey communities (Chiaverini et al., 2025). In line with the habitat productivity hypothesis, this could lead to reduced HR sizes, independently of competition (productivity‐driven HR contraction hypothesis).

Lastly, human population density could limit space use by increasing the likelihood of human–wildlife conflict (Inskip & Zimmermann, 2009), while broader human pressures linked to infrastructure contribute to movement constraints through habitat degradation, fragmentation and fear (Tucker et al., 2018). Thus, we expected HR size to decrease with increasing anthropization (Hirt et al., 2021). Similarly, croplands and pastures modify habitat by reducing available natural resources (Boron et al., 2020) and altering movement patterns though artificial barriers like fencing (McInturff et al., 2020). While major roads can also act as artificial HR barriers (Riley et al., 2006) for some individuals, others may expand their ranges when roads are within their borders (Poessel et al., 2014). Finally, we highlight that all these hypotheses are not mutually exclusive and that interactions between factors might also lead to more complex patterns of variation in HR size (Table 1).

## 2 MATERIALS AND METHODS

### 2.1 Data collection of individual home range sizes in felids

**FIGURE 1:**
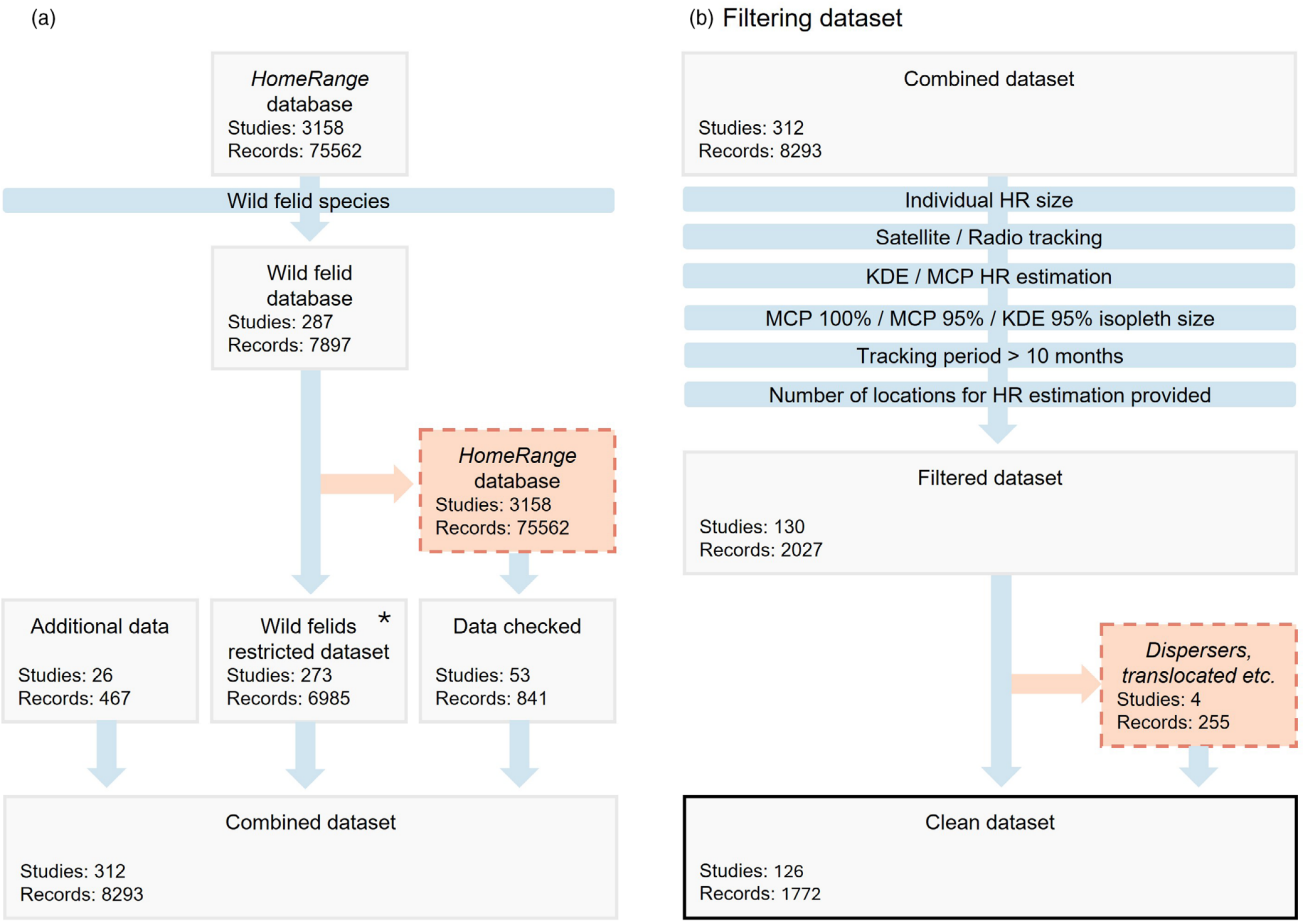
Schematic representation of assembling and filtering steps. (a) Assembling dataset. (b) Filtering dataset. *One excluded study (*n* = 26) from ‘Wild felids’ is now included (only 24 records) in ‘Additional data’ due to updated taxonomy. Another excluded study (*n* = 115) from ‘Wild felids’ has been corrected and is included (now only 38) in ‘Data checked’.

As our study is a meta‐analysis of published data, there was no field work or animal manipulation which required any ethical approval from an animal ethic committee or any licence or permits for fieldwork. Wild felid HR size values were primarily sourced from *HomeRange* (Figure 1), the global database of mammalian home ranges compiled by Broekman et al., 2023. From this database, we extracted 7897 records for species in the *Felidae* family, excluding *Felis catus*, covering studies ranging from 1956 to March 2022. However, some records lacked key information, such as the start and end months of tracking periods and the mean number of locations for some records; therefore, we manually checked and corrected, when possible, by referring to the original papers (Supplement S1). To supplement this dataset, we conducted a comprehensive literature search focusing on the least reported species spanning peer‐reviewed papers, master's and PhD theses, secondary sources (e.g. books), and technical reports going over the same timeframe as the initial database. Subsequently, we extended the search beyond this period (April 2022–January 2024) to include more recent studies, conducting the search on *Web of Science* and *Google Scholar* to identify additional records (Supplement S2).

Finally, the database we assembled consists of annual individual HR size estimations along with detailed information on study duration, geographical location, individual characteristics (e.g. sex, age, body mass), tracking method (TM), HR estimation method (HRM), isopleth size and the number of spatial data points included in the HR estimate. Kernel density estimation (KDE) and minimum convex polygon (MCP) are the most used methods to estimate HR size in mammals, typically using 95% or 100% isopleths, which correspond to the area containing a given percentage of an animal's recorded locations. Radiotelemetry (RT) and satellite tracking (SAT) are the primary TMs used to collect spatial data. Using *R* version 4.4.2 (R Core Team, 2024), we filtered the data (Figure 1) based on TM, HRM, isopleth size, study period and the availability of data for methodological factors, along with additional criteria detailed in Supplement S3. To reduce heterogeneity without decreasing sample size, we standardized isopleth size for MCP estimates by scaling from MCP 95% to MCP 100%, thereby maintaining comparability across all HR size metrics (Rodríguez Recio et al., 2022; Supplement S4, Figure S1) eventually using only MCP 100% records, either observed or predicted, alongside KDE 95% records.

### 2.2 Data collection of life‐history traits (intrinsic factors) and methodological confounders

Sex and age class of each cat was typically provided by each study, although missing data occasionally occurred (see Section 3). Because individual body mass was not consistently available, we assigned each individual the mean adult body mass (ABM) for its sex at the species level, ensuring that sexual dimorphism was accounted for. For species without sex‐specific data, overall adult body mass was used instead (Table S1). Additionally, key methodological confounders were annotated such as the HRM, the TM and the mean number of locations (NLOC) used to estimate HR.

### 2.3 Data collection of environmental factors

Environmental factors were selected following previous studies that used them to reflect similar ecological or anthropogenic processes (see Table 1 for hypotheses). To capture the effect of the ecological factors on felids' space use, we used net primary productivity (NPP) as a proxy for prey availability (Thompson et al., 2021), felid richness (FR)—the number of felid species present at each location—as a proxy of intraguild competition (Macdonald et al., 2010), and elevation (ELE) as a proxy of prey availability but also for less human disturbance (Snider et al., 2021; Walton et al., 2017).

Additionally, to assess the effect of human disturbances, we used Human Footprint Index (HFI) (Hirt et al., 2021), an accumulation of nine proxies for human pressure on the environment, along with separate predictors such as human population density (HPD) (Snider et al., 2021), road density (RD) (Thompson et al., 2021), croplands (CR) and pastures (PS) (Tucker et al., 2021) in the starting model (see below). All data sources are listed in Table S2.

For each geographical location, environmental factors were calculated within a species‐specific buffer based on the median dispersal distance of each species using the packages *raster* V3.6‐26 (Hijmans, 2023) and *rgdal* V1.6‐7 (Bivand et al., 2023). The species‐specific buffer radius was estimated as the median dispersal distance using the equation from Bowman et al. (2002): 7 × mean_HR^0.5^, where mean_HR is the mean home range size for each species. This approach ensures that the species‐specific buffer sizes align with the spatial scale of ecological processes relevant to each felid species (Table S3).

Temporal variation in environmental factors was accounted for by matching the closest available data from the corresponding study year to each study site. Whenever temporal variation (standard deviation of each covariate across years at a given site) was lower than spatial variation (standard deviation of each factor across sites within the same year), annual fluctuations were considered negligible, so these missing values were imputed using data from the closest available year at the same location. The spatial and temporal accuracy varied across factors, with the coarsest resolution being 0.1° (~11 × 11 km) for NPP and data for felid richness, elevation, road density, croplands, pastures being available for a single year (Table S2).

### 2.4 Data preparation for statistical analysis

To avoid pseudoreplication, we only retained records with an individual Ind_ID either provided by the original papers or manually assigned by us when missing. Some individuals contributed multiple records, for example when HR was estimated using different methods (e.g. MCP, KDE) or over different study periods (annual vs. multi‐annual, or across different years). We accounted for this by including ID as a random intercept in our models (see random effects structure below). Due to the underrepresentation of other age classes (see Section 3), only adult individuals were included in the analysis. ABM was log‐transformed (all log‐transformations were natural logarithm) to deal with its skewed distribution and predictors were scaled; we further excluded records with missing information in any of the previously selected variables. Two studies were excluded prior to analysis because the terms ‘extreme’ and/or ‘exceptionally’ appeared in the study title or abstract: one on the Eurasian lynx (*Lynx lynx*) in Norway and one on the cheetah in Namibia. Additionally, records with extreme HR values (i.e. outliers) identified using the Boxplot function from *car* (Fox & Weisberg, 2019) were removed.

### 2.5 Statistical analysis

Before implementing generalized linear mixed models (GLMMs) using the *glmmTMB* package (Brooks et al., 2017), we checked for multicollinearity among predictors, discarding variables with high correlation (|*r*| > 0.7) (Dormann et al., 2013). We tested for a phylogenetic signal in HR size using Pagel's *λ* method to determine whether closely related species exhibit similar HR sizes based on the molecular phylogeny of felids from Li et al. (2016). This analysis was conducted using *phytools* (Revell, 2024), and the results indicated no significant phylogenetic signal (Table S4). However, we included a nested random intercept (1|Genus/Species/ID) to account for potential phylogenetic inertia.

To control for methodological differences, we included TM, HRM and NLOC as fixed effects; since NLOC had a skewed distribution, we log‐transformed it prior to analysis. HRM and TM were treated as fixed effects because each is a factor with only two levels (HRM: MCP, KDE; TM: SAT, RT), which is too few to model as random effects.

We tested the relationship of the covariates and some of their biologically relevant two‐way interactions (Table 1) with HR size using GLMM with a log link and gamma error structure, starting from the umbrella model below (see Section 3):

$$\begin{array}{c} \mathrm{HR}\;\text{size}\sim \mathrm{ABM}+\mathrm{SEX}+\mathrm{HRM}+\mathrm{TM}+\text{NLOC}+\mathrm{ELE}+\mathrm{NPP}+\mathrm{FR}+\mathrm{RD}\\ \;+\mathrm{RD}:\mathrm{ABM}+\mathrm{CR}+\mathrm{CR}:\mathrm{ABM}+\mathrm{PS}+\mathrm{PS}:\mathrm{ABM} \end{array}$$

We compared and selected the best random effects structure for taxonomy as we included a nested random intercept (1|Genus/Species/ID) to account for potential phylogenetic inertia (see Section 3) and a random intercept effect of Study ID to control for unequal numbers of HR size estimates from different studies. The initial random structure was (1|Genus/Species/ID) + (1| Study ID) and the best random effect structure was selected based on the lowest Akaike information criterion (AIC) and retained in the model for the following analyses. We then conducted model simplification via the dredge function of the *MuMIn* package (Bartoń, 2023) using AICc for model comparison. We averaged the models with the lowest AIC (ΔAIC <2) via the *model.avg* function from the same package. Fixed effects were retained based on their significance in the conditional averaged model. The final model included these significant fixed effects, along with random slopes by *Species* for the significant environmental factors and their interactions. This approach allowed for variability across species and helped control for type I error in our estimates (Schielzeth & Forstmeier, 2009). Additionally, a random slope was added for NLOC to account for differences among species. Results are reported as mean estimates ± standard error unless otherwise stated. We calculated marginal (*R* ^2^_m_) and conditional (*R* ^2^_c_) coefficients of determination for the GLMM, using the *r.squaredGLMM* function from the *MuMIn* package, to quantify the variance explained by fixed effects alone and by the full model (fixed and random effects combined), respectively. We assessed the fit of the final model using the diagnostic plots (Figure S2) and tested for spatial autocorrelation with Moran's *I* test (Table S5) from the *DHARMa* package (Hartig, 2022). We calculated marginal responses, that is, mean estimated values for each predictor assuming all other predictors at the constant level, accounting for the random effects structure using *ggeffects* (Lüdecke, 2018). To assess the robustness and the contribution of each species to the pattern we have found, we conducted a leave‐one‐out (LOO) sensitivity analysis on the final model (Supplement S5; Table S6; Figure S3).

## 3 RESULTS

### 3.1 Dataset overview

**FIGURE 2:**
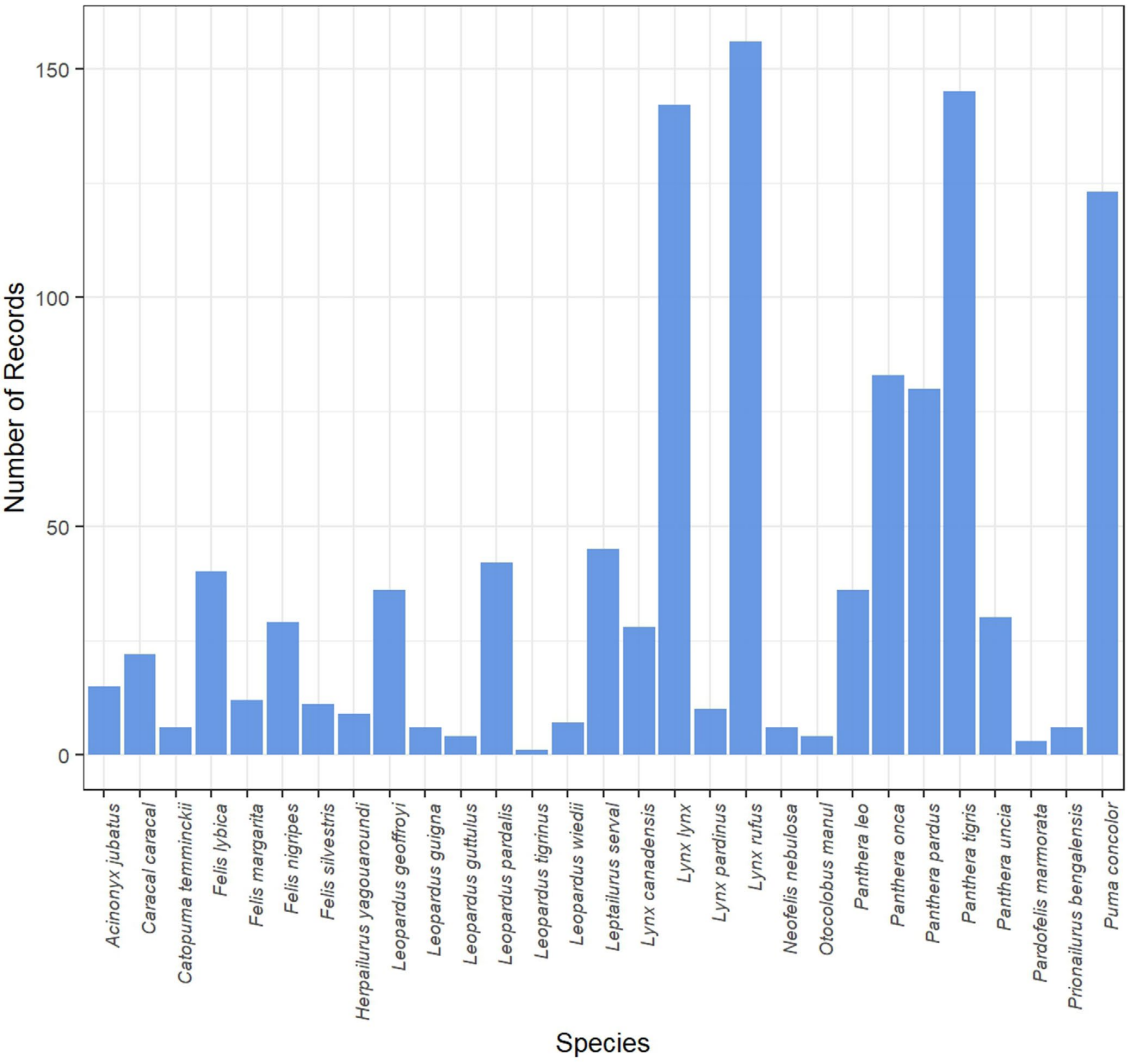
Histogram of records by species present in Home Range dataset used for modelling.

In the dataset, 89% of records were stated as adults, 8% as subadults and 3% as juveniles; therefore, only adult HR sizes (*n* = 1160) were retained. Records with missing values (*n* = 7, all for Sex) and outliers (*n* = 16) were removed. The final dataset for the GLMM analysis consisted of 1137 records from 582 adult individuals across 29 species, collected from 114 studies, with a female‐to‐male sex ratio of 0.58. The median (IQR) number of records per species was 22 (6 to 42), with representation ranging from a minimum of one record for species such as *Leopardus tigrinus* to a maximum of 156 records for *Lynx rufus* (Figure 2; Table S7).

**FIGURE 3:**
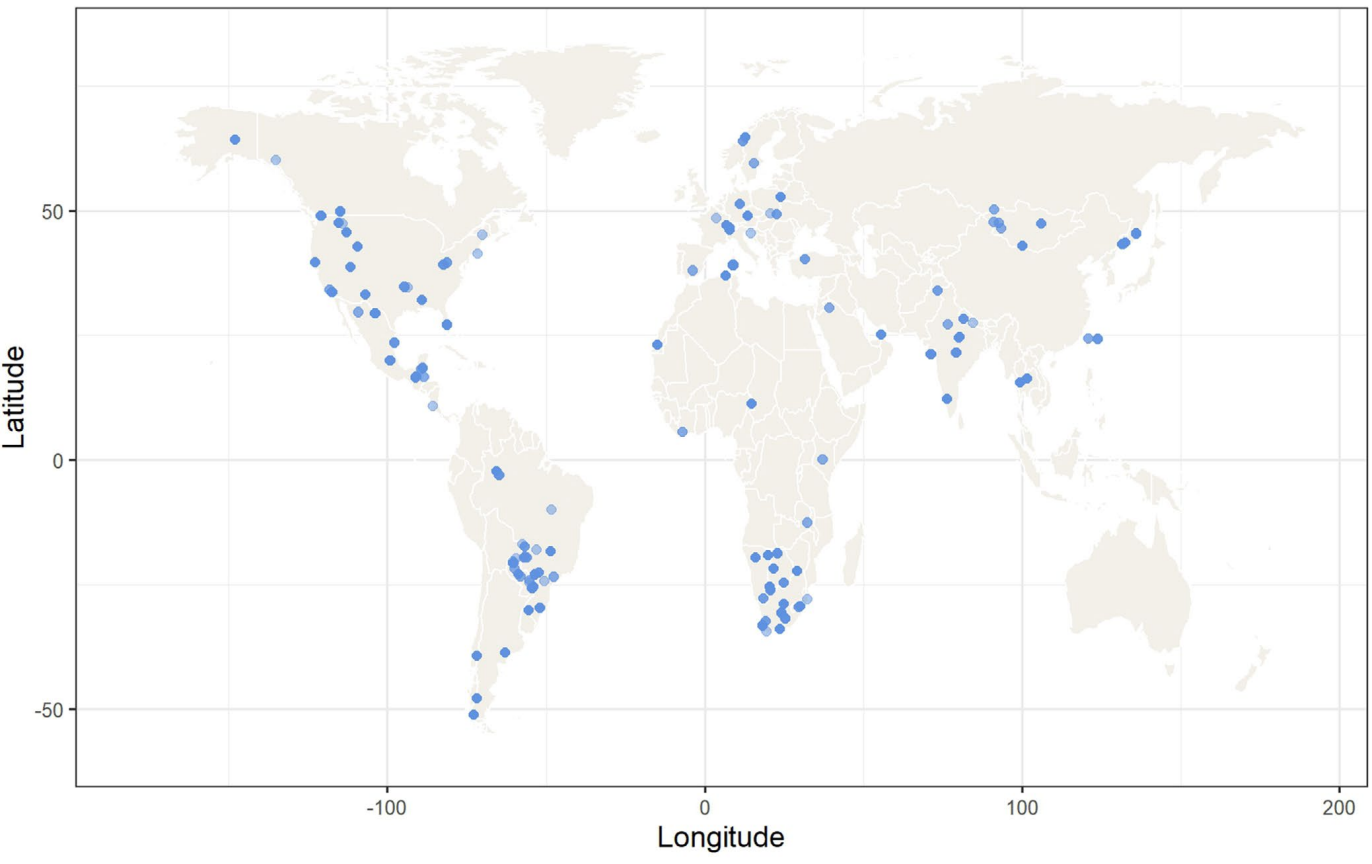
Global distribution of study sites used in the Home Range size analysis.

Our study covered a total of 159 unique study sites in 38 different countries across Africa (*n* = 212), Asia (*n* = 211), Europe (*n* = 219), South America (*n* = 166) and North America (*n* = 329) (Figure 3).

Before analysis, HPD and HFI were removed due to strong correlations with Road density and Croplands (Figure S4).

### 3.2 Home range analysis

The best random effects structure for HR analysis included ID nested within Species and Study ID as random intercepts (Table S8). The best averaged conditional model included significant effects for ABM, Sex, HRM, NLOC, Elevation, NPP, Felid richness, Croplands, Croplands:ABM, Pastures and Pastures:ABM (Table 2; Table S9).

**TABLE 2:** Summary of the results from the model selection for HR dataset based on Akaike information criterion (AICc).

Model	df	logLik	AICc	ΔAICc	Weight
ABM + SEX + HRM + NLOC + ELE + NPP + FR + CR + CR:ABM + PS + PS:ABM	16	−5750.304	11,533.1	0.00	0.399
ABM + SEX + HRM + NLOC + ELE + NPP + FR + CR + CR:ABM	14	−5753.079	11,534.5	1.44	0.194
ABM + SEX + HRM + TM + NLOC + ELE + NPP + FR + CR + CR:ABM + PS + PS:ABM	17	−5750.205	11,535.0	1.86	0.157
ABM + SEX + HRM + NLOC + ELE + NPP + FR + RD + CR + CR:ABM + PS + PS:ABM	17	−5750.295	11,535.1	2.04	0.143
INTERCEPT	5	−5929.288	11,868.6	NA	NA

*Note*: Models with ΔAICc of less than two are listed in black and are used for model averaging.

In the final model (Table 3), after including random slopes (see Section 2), fixed effects alone explained 42% of the variance (*R* ^2^_m_ = 0.42), while the full model including random effects explained 96% of the total variance (*R* ^2^_c_ = 0.96).

**TABLE 3:** Parameter estimates of the final model for home range size prediction after model averaging and addition of random slopes for all environmental covariates, CR:ABM and PS:ABM interactions and NLOC.

Parameter	Estimate	SE	*Z*‐value	*p*‐value
INTERCEPT	3.911	0.223	17.560	<2e−16***
Elevation	0.018	0.155	0.117	0.907
NPP	−0.369	0.068	−5.405	6.48e−08***
Croplands	−0.499	0.143	−3.482	4.97e−04***
Pastures	−0.160	0.070	−2.277	0.023*
Felid richness	−0.237	0.100	−2.363	0.018*
Sex (M)	0.514	0.067	7.665	1.79e−14***
ABM	0.938	0.161	5.832	5.48e−09***
HRM (MCP)	0.184	0.033	5.650	1.60e−08***
NLOC	0.214	0.046	4.679	2.88e−06***
Croplands:ABM	0.143	0.103	1.388	0.165
Pastures:ABM	0.088	0.064	1.376	0.169

* *p* < 0.05,

*** *p* < 0.001.

**FIGURE 4:**
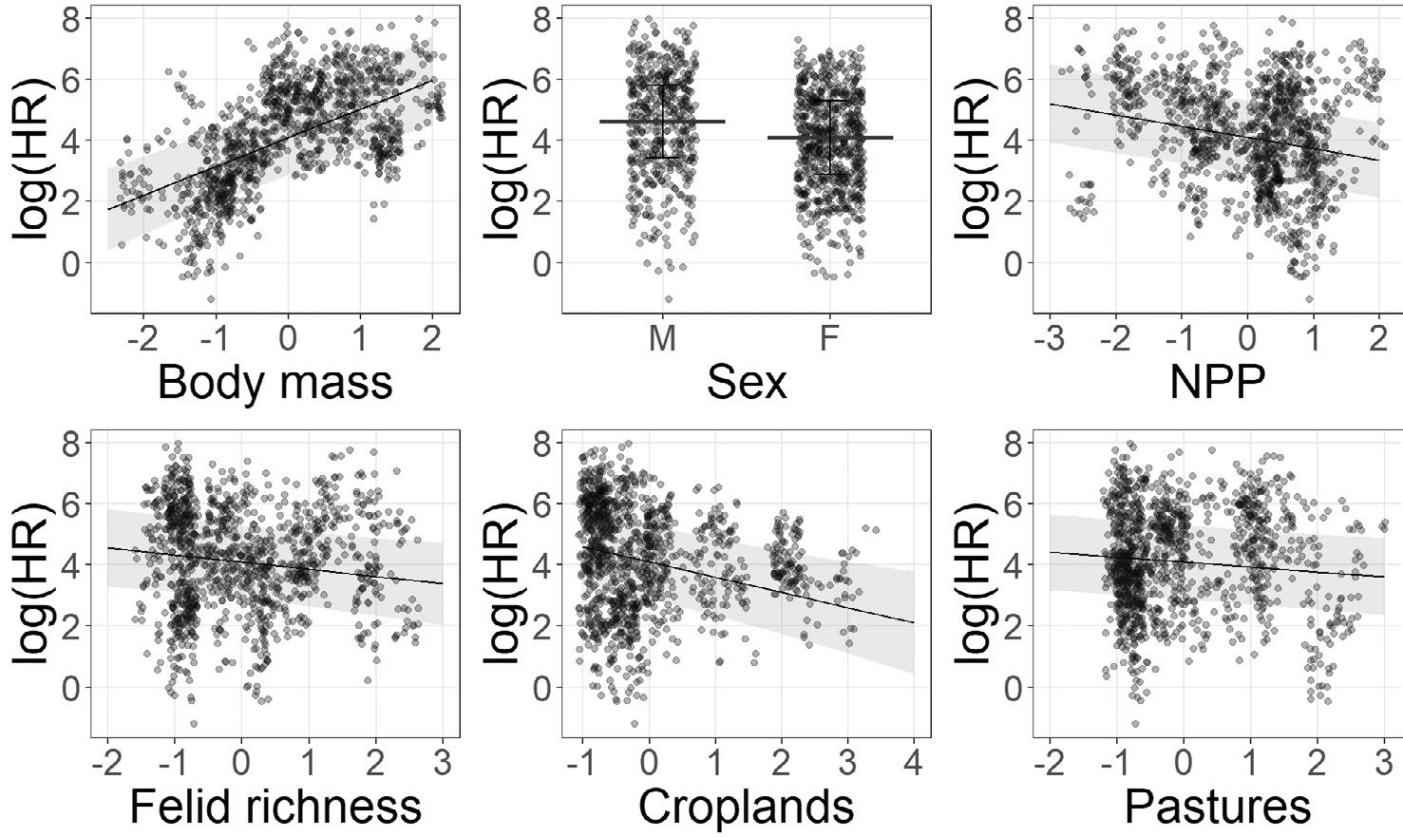
Marginal responses of HR to ABM, Sex, NPP, Felid richness, Croplands and Pastures calculated from GLMM with random intercept for taxonomy and Study ID and random slopes by Species for environmental variables, Croplands:ABM and Pastures:ABM interactions and NLOC. Lines indicate predicted response, and grey area indicates 95% prediction intervals.

Among the random effects, most variance was explained by Species, followed by Study ID and individual differences within species, while random slopes for ecological and anthropogenic predictors contributed little additional variation (except for Croplands) (Table S10). We found that intrinsic traits had a clear effect on space use: There was very strong evidence that ABM was positively associated with HR size (0.94 ± 0.16; *p* < 10^−8^) and that males tended to have larger HRs than females (0.51 ± 0.07; *p* < 10^−13^). Additionally, some methodological confounding factors also affected HR size, with MCP‐based estimates being larger than KDE‐based ones (0.18 ± 0.03; *p* < 10^−7^) and HR size increasing with increasing NLOC (0.21 ± 0.05; *p* < 10^−5^). Among the ecological covariates, there was very strong evidence that higher NPP was associated with smaller home ranges (−0.37 ± 0.07; *p* < 10^−7^) and moderate evidence that higher felid richness (−0.24 ± 0.10; *p* = 0.02) also decreased HR size. Finally, HR size tended to decrease with increasing anthropization, with very strong evidence for Croplands (−0.50 ± 0.14; *p* < 10^−3^) and moderate evidence for Pastures (−0.16 ± 0.07; *p* = 0.02) (Figure 4). There was no evidence for an effect of Elevation or the interaction terms Croplands:ABM and Pastures:ABM on HR size once random slopes were included in the model.

### 3.3 Sensitivity analysis

Of the 29 LOO models, 24 converged. Convergence failures were not linked to removing species with extreme sample sizes or HR values (Figure S3). Among the converged models, 83.3% matched the full model, rising to 91% under a less stringent criterion (Table S6).

## 4 DISCUSSION

This study investigated the global predictors of Home Range (HR) size in felids, a taxon of major conservation concern (Dickman et al., 2015), taking advantage of the large amount of HR size data already available for this group in the literature (Broekman et al., 2023), which we complemented with nearly 20% additional records.

We modelled the effects of a comprehensive set of predictors of HR size with intrinsic traits (e.g. body mass and sex), ecological factors (e.g. felid richness, elevation, productivity) and human‐impact factors (e.g. road density, agricultural lands) while also controlling for methodological confounders (e.g. tracking and estimation method, number of locations) to explain the pattern of space use variability across the *Felidae* family.

Our results identified several key drivers of space‐use. As expected, intrinsic factors shaped HR size: body mass was positively correlated with space use, males occupied larger HRs than females. Of the three methodological confounders considered, only the HRM and the NLOC significantly influenced HR estimates. Among extrinsic factors, net primary productivity, felid richness, croplands and pastures all reduced HR size as expected, whereas road density and elevation had no significant effect. The sensitivity analysis confirmed that our model was robust to removing any single species, including the most represented and those with lowest or highest HR values.

Variation in HR size was primarily driven by differences in baseline HR size among species, reflecting inherent ecological and life‐history differences. Additional variation arose from study‐level effects, with a smaller contribution from individual differences within species. In contrast, species‐specific slopes contributed little, indicating similar responses to ecological and anthropogenic factors.

### 4.1 Intrinsic factors and methodological confounders

HR size increased with body mass, consistent with the general expectation that larger individuals require extensive territories to meet their energy needs (Tucker et al., 2014). Males, as expected, occupied larger HRs than females, reinforcing the well‐established sexual dimorphism in space use among felids and beyond taxa, likely due to males' need to cover larger areas for mating opportunities, a common trait among polygynous and solitary species (Duncan et al., 2015; Rodríguez Recio et al., 2022).

Although not the primary focus, the influence of methodological confounders such as HRM and NLOC on HR size demonstrates the need for careful reporting, standardization and consideration of methodological differences when comparing space use metrics across studies and species.

### 4.2 Ecological factors

Net primary productivity reduced HR size, which is consistent with our initial predictions and supports the habitat productivity hypothesis, which proposes that a resource‐rich environment reduces the need for an animal to roam large distances to meet their energy needs, thereby decreasing optimal HR size. Although we lack independent data linking vegetation productivity directly to herbivore density, it is a reasonable assumption that such a relationship exists. Our results demonstrate that more productive habitats, implying greater prey availability, allow felids to maintain smaller HRs. This pattern, previously observed either at the species and population level (e.g. Eurasian lynx: Herfindal et al., 2005; leopard: Thompson et al., 2021; jaguar: Snider et al., 2021) or at the higher order of *Carnivora* (Broekman et al., 2024; Duncan et al., 2015; Hirt et al., 2021), also holds across a wide range of felid species and at a global scale.

Our results show that felid richness reduces HR size, indicating that individuals occupy smaller home ranges in areas with more felid species. This pattern is inconsistent with competition‐driven HR expansion and instead supports mechanisms leading to HR contraction, either through competition‐driven HR contraction or productivity‐driven contraction. The negative relationship between felid richness and HR size is best explained in our study by coexistence mechanisms that reduce competition, allowing individuals in species‐rich communities to occupy smaller, more efficiently used ranges. One key process underlying this pattern is niche differentiation, which mitigates interspecific competition by partitioning available resources (Steinmetz et al., 2021). In carnivore guilds, such differentiation often occurs along the dietary niche axis, which strongly influences HR size because the diversity of prey exploited determines the area it must cover to meet energetic needs. Under interspecific competitive conditions, larger felids tend to adopt broader, more generalist diets, whereas smaller felids become more specialized. For example, Vogel et al. (2019) reported that dominant species such as lions and leopards typically exploit a wider range of prey, whereas subordinate competitors like cheetahs and wild dogs (*Lycaon pictus*) focus on fewer, more specific prey types (Vogel et al., 2019). Despite occurring in opposite directions, both shifts are associated with a reduction in HR size in terrestrial mammals (Huang et al., 2021). Large generalists can maintain smaller HRs than large specialists, who might need to travel farther to find their preferred. Small specialists, focusing on one reliable prey type, could meet their needs within a limited, predictable area, resulting in smaller HRs than small generalists (Huang et al., 2021). Thus, increasing felid richness may drive a dual response—dominant species expand diet breadth, subordinate species compress it—resulting in smaller HRs overall. Individual behavioural adaptations can further decrease HR size through spatial efficiency when other species are present. Felids can reuse habitual travel routes within their home ranges (Fagan et al., 2025), which allows them to forage efficiently by minimizing unnecessary movement. This spatial optimization provides a plausible explanation for how competition‐driven niche differentiation can reduce HR size. Overall, these patterns suggest that competition among coexisting felids drives behavioural adjustments—such as dietary niche differentiation and optimized space use—that translate into smaller HRs. Thus, HR contraction emerges as a response to interspecific competition, facilitating coexistence in species‐rich communities. Although our global analysis supports HR contraction with increasing species richness, some local‐scale studies have reported the opposite pattern, where interspecific competition leads to HR expansion. For instance, Kumar et al. (2019) documented expansion of HR size in the subordinate species; leopards, which are three times smaller than tigers, increased their HR size in their presence. This suggests that additional factors may also play a role such as population density of each cat species (tiger densities were notably high in that study). Furthermore, Oliveira et al. (2010) showed that in areas with greater human disturbance, smaller sympatric felids (*Leopardus wiedii*, *Leopardus tigrinus*, *Herpailurus yagouaroundi*) adjusted to avoid the larger ocelots (*Leopardus pardalis*) by increasing their HR size, illustrating how human impacts can exacerbate intraguild competition. These findings illustrate that the direction and magnitude of competitive effects are context‐dependent, influenced by local predator densities, resource distributions and anthropogenic pressures. In our dataset, NPP and felid richness were negatively correlated, providing little support for productivity‐driven HR contraction. However, because predator richness is often more closely linked to prey richness than to overall productivity (Sandom et al., 2013), regions with a diverse prey base could still allow felids to meet their energetic needs within smaller ranges. Since we lack direct prey richness data, this mechanism cannot be explicitly tested here, leaving it open for further investigation. In conclusion, higher felid richness was associated with smaller home ranges, likely reflecting both ecological and behavioural optimization processes, such as dietary niche differentiation and efficient space use, that facilitate coexistence. While local competition can sometimes expand HRs, at a global scale, these strategies appear to allow multiple species to share space more efficiently suggesting that the underlying mechanisms linking home range to felid richness are not mutually exclusive and likely vary with spatial scale. Although we did not find statistical support for a quadratic effect, the observed pattern may be more complex than a simple linear relationship and should be further explored.

### 4.3 Anthropization factors

Human pressures (measured by HFI) were previously found to be a significant predictor of HR size in terrestrial mammals (Broekman et al., 2024) and carnivores (Hirt et al., 2021). While we could not include HFI directly in our analysis, we incorporated its components and found that land‐use changes, specifically Croplands and Pastures, significantly reduced HR size. The influence of agricultural land on HR size could be explained by either restricted movement (e.g. associated roads or fences), increased resource availability—whether through depredation of livestock (Khorozyan et al., 2015) or higher prey densities in croplands (Curveira‐Santos et al., 2021)—or a combination of both; however, determining which mechanism is at play is beyond the scope of our study, but future studies should investigate this point deeper. Our findings suggest thus that increasing agricultural land density reduces felid HR size, this effect echoes broader patterns of agricultural landscapes being unsuitable habitats for felids (Caruso et al., 2015; Dickson & Beier, 2002) and supports previous studies which have identified habitat loss and degradation (IUCN, 2024; Ripple et al., 2014), largely driven by cropland expansion (Tang et al., 2021), as primary threats to felids at the global scale. Large‐scale monoculture and extensive grazing pastures fragment and reduce natural habitats for felids (Caruso et al., 2015). In addition, when unsuitable habitats cannot be easily avoided (Boron et al., 2020; Dickson & Beier, 2002), they may also become sources of human‐felid conflicts, which can in turn influence space and time use strategies. For example, in Nepal, leopard attacks on livestock occur close to forest edges and livestock enclosures (Chaudhary et al., 2025), suggesting limited spatial separation between anthropogenic and natural spaces increases depredation and by extent retaliatory killings of felids (Bano et al., 2021). Similarly, tiger activity decreased when human activity increased (Carter et al., 2012). In such contexts, the preference of felids for easily accessible prey like sheep, goats and pigs could lead to spatial contraction of HRs in highly anthropized landscapes where natural prey is scarce but livestock is abundant (Khorozyan et al., 2015), ultimately generating more human–wildlife conflicts. These findings suggest that while broad measures of human pressure may be useful across diverse mammalian taxa, specific landscape modifications such as agricultural expansion may be more relevant for understanding space use in felids.

### 4.4 Limits

Due to data availability, our study focused exclusively on HR size in adult felids. While this constraint minimizes the risk of including unstable HR sizes (as adults tend to disperse less than subadults), it leaves a gap in understanding how other age classes respond to the same environmental factors. For example, it is not unreasonable to assume the subadult cats might place their HR into suboptimal habitat to reduce the competition with the adults (Morrison et al., 2015).

Additionally, due to limited individual body mass records in our database, we used mean ABM as a proxy, which failed to capture intraspecific variability in body mass; this limitation might obscure specific behaviours linked to individual body mass and mask its more subtle interactions with environmental factors, potentially explaining why they were not significant in our analysis. We hence recommend that whenever HR sizes are reported, the body mass of each individual is aptly provided in order to reduce this bias.

Species‐specific buffers were estimated from median dispersal distances calculated using mean HR, which does not account for sex differences and may slightly overestimate buffers for females. Nevertheless, we consider this a reasonable approximation at the global scale, given the relatively balanced sex ratio in our dataset (0.56).

Felid richness emerged as a predictor of HR size, suggesting potential effects of interspecific interactions on space use at broad spatial scales. In our study, felid richness was calculated within species‐specific dispersal buffers, which likely capture regional coexistence patterns rather than fine‐scale intraguild competition. Understanding local space use requires considering the full guild present at a given time. However, our dataset did not include enough simultaneous HR estimates for multiple species at the same sites nor the identities of co‐occurring species, preventing us from directly testing the direction or magnitude of competition at the local level. While our results hint at a global signal of interspecific effects, disentangling local competitive dynamics would require fine‐scale multi‐species tracking data.

Despite a large database, there was wide variability in the number of HR records across species (Figure 2; Table S5), with larger species overrepresented and smaller ones underrepresented or absent (Anile & Devillard, 2016; Macdonald et al., 2010). Future research should focus on the HR dynamics of smaller and less studied species, thereby improving representation and deepening our understanding of felid spatial ecology.

## 5 CONCLUSIONS

In conclusion, this study uncovers the multifaceted drivers of felid space‐use, emphasizing the importance of considering intrinsic (Body mass and Sex) and extrinsic (net primary productivity, felid richness, croplands and pastures) factors, while accounting for methodological confounding factors (HRM, TM and number of locations) to provide reliable conservation measures. Our findings highlight the vulnerability of felids to habitat modifications (Zanin et al., 2015), especially agricultural land use, and the need for evidence‐based conservation strategies that address both ecological and anthropogenic pressures to ensure the long‐term survival of these species (Sutherland et al., 2004). This could involve continuing to develop protected areas around the world where human activities could be curbed through sensible management of land use and minimization of anthropogenic stressors. One of our main findings is the negative relationship between felid richness and home range size, which suggests that spatial contraction can facilitate coexistence among multiple species. At the same time, this compression could create stress, as reduced HRs could also lead to suboptimal space use, potentially affecting population persistence through competitive processes. Even if species appear to cope behaviourally with the presence of others, this compression could still negatively impact their overall fitness—which is particularly concerning given that many felid species are already endangered. This calls for perhaps more careful management of protected areas, particularly fenced ones, by considering the community composition when assessing habitat suitability and avoiding the introduction of too many felid species into a single area in order to allow sympatric processes to take place without any detrimental effects. Felids often serve as umbrella species, that is their conservation benefits numerous co‐occurring species. Thus, through a better understanding of their space use patterns, we can enhance conservation strategies to ensure the survival and ecological functionality of felid populations, ultimately benefiting entire ecosystems (Tossens et al., 2025).

## AUTHOR CONTRIBUTIONS

Stefano Anile and Sébastien Devillard designed the study, Stefano Anile and Arthemis Moraru collected the data, Stefano Anile, Arthemis Moraru and Sébastien Devillard analysed the data, Arthemis Moraru drafted the first version of the manuscript, and all authors equally revised it. Our study was a global meta‐analysis and was based on an analysis of secondary data rather than primary data. As such, there was no local data collection.

## CONFLICT OF INTEREST STATEMENT

The authors declare no conflict of interest.

## Supporting information


**Supplement S1:** Manual check and corrections of *HomeRange* database.
**Supplement S2:** Web of Science and Google Scholar search queries for home range estimation in felids.
**Supplement S3:** Data filtering.
**Supplement S4:** Data scaling from MCP 95% to MCP 100%.
**Supplement S5:** Sensitivity analysis of the home range model.
**Figure S1:** Comparison of home ranges estimated with MCP 100% vs. MCP 95%.
**Figure S2:** Residual diagnostics plot made with DHARMa for final HR model.
**Figure S3:** Home range size (km^2^) by species ordered by median Home range size.
**Figure S4:** Correlation plot for HR dataset.
**Table S1:** Source and type of mean adult body mass (ABM) data used for each felid species.
**Table S2:** Overview of global databases used for extracting environmental factors.
**Table S3:** Median dispersal (km) as buffer radius and buffer size (km^2^) for each species.
**Table S4:** Phylogenetic signal for home range size assessed using Pagel's *λ* method.
**Table S5:** Spatial autocorrelation for home range size assessed using Moran's *I* test.
**Table S6:** Summary of the leave‐one‐out sensitivity analysis.
**Table S7:** Number of records by species present in Home Range (HR) dataset used for modelling. The total number of records is presented in bold.
**Table S8:** Anova table comparing random effect structures in initial GLMM model for HR dataset (a) with or without Genus and (b) with or without Study ID.
**Table S9:** Parameter estimates for home range size prediction after model averaging (conditional average reported).
**Table S10:** Variance components of random effects in the final GLMM for HR size.

## Data Availability

Data and codes are publicly available from the Zenodo repository https://doi.org/10.5281/zenodo.18315113 (Moraru et al., 2026).
