# Supplementary material for: Global determinants of home range sizes in felids: Evidence of human disturbance impact
Source: J Anim Ecol. 2026 Feb 4;95(4):697–711. doi: 10.1111/1365-2656.70227 (PMC13039275; doi:10.1111/1365-2656.70227)
Supplement: Supplementary file 1 — Supplement S1: Manual check and corrections of HomeRange database. Supplement S2: Web of Science and Google Scholar search queries for home range estimation in felids. Supplement S3: Data filtering. Supplement S4: Data scaling from MCP 95% to MCP 100%. Supplement S5: Sensitivity analysis of the home range model. Figure S1: Comparison of home ranges estimated with MCP 100% vs. MCP 95%. Figure S2: Residual diagnostics plot made with DHARMa for final HR model. Figure S3: Home range size (km2) by species ordered by median Home range size. Figure S4: Correlation plot for HR dataset. Table S1: Source and type of mean adult body mass (ABM) data used for each felid species. Table S2: Overview of global databases used for extracting environmental factors. Table S3: Median dispersal (km) as buffer radius and buffer size (km2) for each species. Table S4: Phylogenetic signal for home range size assessed using Pagel's λ method. Table S5: Spatial autocorrelation for home range size assessed using Moran's I test. Table S6: Summary of the leave‐one‐out sensitivity analysis. Table S7: Number of records by species present in Home Range (HR) dataset used for modelling. The total number of records is presented in bold. Table S8: Anova table comparing random effect structures in initial GLMM model for HR dataset (a) with or without Genus and (b) with or without Study ID. Table S9: Parameter estimates for home range size prediction after model averaging (conditional average reported). Table S10: Variance components of random effects in the final GLMM for HR size. [file JANE-95-697-s001.zip › jane70227-sup-0001-FigureS1-S4-TableS1-S10@Supporting information_HR_felids.docx]

**Supporting information**

[Supplement S1: Manual check and corrections of *HomeRange* database 2](#_Toc213616487)

[Supplement S2: Web of Science and Google Scholar search queries for home range estimation in felids. 3](#_Toc213616488)

[Supplement S3: Data filtering 4](#_Toc213616489)

[Supplement S4: Data scaling from MCP 95% to MCP 100% 5](#_Toc213616490)

[Supplement S5: Sensitivity analysis of the home range model 13](#_Toc213616491)

[Figure S1: Comparison of home ranges estimated with MCP 100% vs MCP 95%.. 6](#_Toc213616514)

[Figure S2: Residual diagnostics plot made with DHARMa for final HR model. 11](#_Toc213616515)

[Figure S3: Home range size (km²) by species ordered by median Home range size. 17](#_Toc213616516)

[Figure S4: Correlation plot for HR dataset. 19](#_Toc213616517)

[Table S1: Source and type of mean adult body mass (ABM) data used for each felid species. 7](#_Toc213616544)

[Table S2: Overview of global databases used for extracting environmental factors. 8](#_Toc213616545)

[Table S3: Median dispersal (km) as buffer radius and buffer size (km2) for each species. 9](#_Toc213616546)

[Table S4: Phylogenetic signal for home range size assessed using Pagel’s λ method. 10](#_Toc213616547)

[Table S5: Spatial autocorrelation for home range size assessed using Moran’s I test. 12](#_Toc213616548)

[Table S6: Summary of the leave-one-out sensitivity analysis. 16](#_Toc213616549)

[Table S7: Number of records by species present in Home Range (HR) dataset used for modelling. The total number of records is presented in bold. 18](#_Toc213616550)

[Table S8: Anova table comparing random effect structures in initial GLMM model for HR dataset a) with or without Genus and b) with or without Study ID. 20](#_Toc213616551)

[Table S9: Parameter estimates for home range size prediction after model averaging (conditional average reported). 21](#_Toc213616552)

[Table S10: Variance components of random effects in the final GLMM for HR size 22](#_Toc213616553)

#### Supplement S1: Manual check and corrections of *HomeRange* database

We identified 881 relevant, pre-selected records *that required manual checking* because of missing information. These records were pre-screened using a subset of the filtering guidelines detailed in Material and Methods and later in Supplement S3, to avoid manually reviewing records that would ultimately be excluded. Whenever possible, we referred to the original papers to verify, correct, or complete missing data. Following this process, 481 records were modified (e.g., missing values filled, minor corrections made), 72 records were removed due to unresolved inconsistencies, while 32 records were added when estimates were found in the supplementary data. We also updated the taxonomy for 24 records.

Additionally, we excluded seven records from the main database – two from studies with revised taxonomy and five from studies with removed records – as they originated from the previous studies deemed erroneous.

#### Supplement S2: Web of Science and Google Scholar search queries for home range estimation in felids.

We conducted the search on Web of Science using tailored queries for each Felid species using the following parameters:

*(TI = (“Scientific name” OR “Common name*” OR “Old Scientific name”)
 OR AB = (“Scientific name” OR “Common name*” OR “Old Scientific name))*

*AND*

*AB = (“home-range” OR “home range” OR “homeranges” OR “home ranges”
 OR “space use” OR “space-use” OR “polygon” OR “kernel” OR “convex”)
 Dates: 1956-01-01 – 2024-01-08*

We further expanded our search using Google Scholar to identify additional records that were not present in the Web of Science database:

*1^st^ query: “Scientific name” ”Home range”*

*2^nd^ query: “Common name” ”Home range”*

*3^rd^ query: “Old version of Scientific name” “Home range”*

*Dates: 1956 – 2024*

This complementary search wasn’t exhaustive as it specifically targeted studies reporting individual HRs estimated using MCP or KDE methods and based on radio or satellite tracking, and the availability of data (tracking period and mean number of locations provided). From this we gathered 467 new partially filtered records.

#### Supplement S3: Data filtering

We included data from individual cats tracked by Satellite (GPS) or radio-tracking (VHF/UHF) in the wild and only records utilizing KDE and MCP methods for estimating HR size with isopleths of 100%, 95%, and 50% but excluded KDE 100% records due to limited sample size (*n* = 43) and insufficient equivalents with KDE 95% to allow scaling between isopleths. We included in our data only reports of annual HRs or equivalents, ensuring a tracking period of more than 10 months and availability of the mean number of locations used. We removed duplicates (i.e. the same individuals described in two or more studies), as well as records originating from individuals considered as dispersers, shifters, translocated and reintroduced individuals. However, translocation/reintroduction events older than 12 months were accepted into the dataset, as these individuals were considered to have adjusted to their new environment and established stable HRs (Figure 1).

#### Supplement S4: Data scaling from MCP 95% to MCP 100%

At this point, our dataset was made up of 354 HRs estimated with MCP 95% and 653 with MCP 100%. To ensure a robust comparison between scaled records and non-scaled records, as well as to investigate the feasibility of scaling these estimates together, we filtered the data to include only instances where the same individual was tracked during the same period and the HR was estimated using both MCP 100% and MCP 95%. Using these criteria, we narrowed the dataset down to 106 individuals spanning 8 species (Figure S1). A linear mixed-effects model was fitted using the *lme4* package to predict MCP 100% home range estimates from MCP 95% estimates, incorporating nested random intercepts for Study Period, Individuals and Species to account for the data structure: *log( HR_MCP100_ ) ∼ log( HR_MCP95_ ) + ( 1|Species / Individuals / Study Period ).* As expected, we found a linear relationship between the 100% and 95% MCP home ranges in the dataset (*R^2^* = 0.97), thus, we used the linear relationship to predict MCP 100% from observed MCP 95%, eventually using only MCP 100% records, either observed or predicted, alongside KDE 95% records.

**Figure S1**


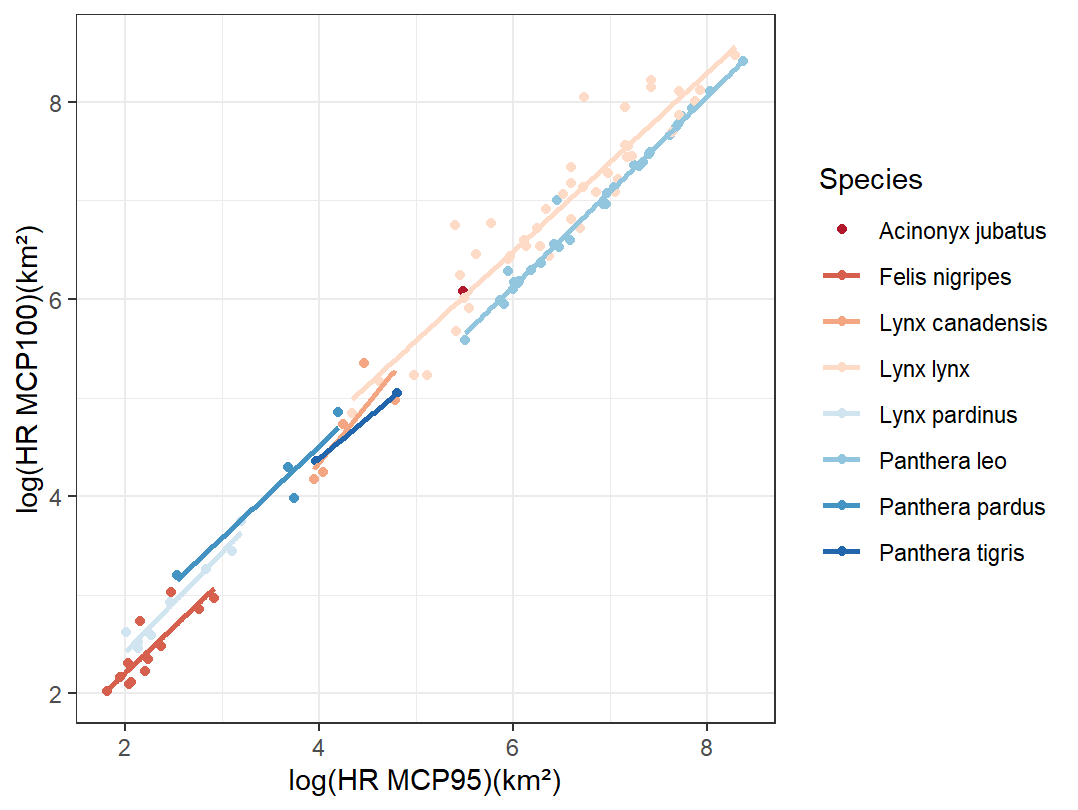


Figure S1: Comparison of home ranges estimated with MCP 100% vs MCP 95%. Only records estimated with both MCP 100% and MCP 95% are included (n=106).

**Table S1**

Table S1: Source and type of mean adult body mass (ABM) data used for each felid species.

| **Species** | **ABM type** | **Source** |
| --- | --- | --- |
| *Acinonyx jubatus*, *Caracal caracal*, *Catopuma temminckii*, *Felis margarita*, *Felis silvestris*, *Herpailurus yagouaroundi*, *Leopardus geoffroyi*, *Leopardus guigna*, *Leopardus pardalis*, *Leopardus wiedii*, *Leptailurus serval*, *Lynx canadensis*, *Lynx lynx*, *Lynx pardinus*, *Lynx rufus*, *Neofelis nebulosa*, *Otocolobus manul*, *Panthera leo*, *Panthera onca*, *Panthera pardus*, *Panthera tigris*, *Panthera uncia*, *Prionailurus bengalensis*, *Puma concolor* | Sex-specific | Johnson, Paul & Noonan, Michael & Kitchener, Andrew & Harrington, Lauren & Newman, C & Macdonald, David. (2017). Data used in ‘results’ section. |
| *Felis lybica*, *Felis nigripes*, *Leopardus guttulus*, *Leopardus tigrinus, Pardofelis marmorata* | Non-sex-specific | Pacifici M, Santini L, Di Marco M, Baisero D, Francucci L, Grottolo Marasini G, Visconti P, Rondinini C (2013) Database on generation length of mammals. 5427 data records. |

**Table S2**

*Table S2: Overview of global databases used for extracting environmental factors.*

| **Environmental factors** | **Abbreviation** | **Source** | **Spatial resolution** | **Time period** |
| --- | --- | --- | --- | --- |
| Elevation | ELE | Hijmans RJ, Barbosa M, Ghosh A, Mandel A (2024). geodata: Download Geographic Data. R package version 0.6-2, [https://CRAN.R-project.org/package=geodata](https://cran.r-project.org/package=geodata) | 1 km (30 arc seconds) aggregated from 90 m resolution data | 2000 |
| Net Primary Productivity | NPP | Running, S., Zhao, M. (2021). MODIS/Terra Net Primary Production Gap-Filled Yearly L4 Global 500m SIN Grid V061 [Data set]. NASA EOSDIS Land Processes Distributed Active Archive Center. Accessed 2024-15-10 from<https://neo.gsfc.nasa.gov/view.php?datasetId=MOD17A3H_Y_NPP> | 0.1 degrees (~11x11 km) | 2001:2023 |
| Felid Richness | FR | Center For International Earth Science Information Network-CIESIN-Columbia University, & NatureServe. (2015). Gridded Species Distribution: Global Mammal Richness Grids, 2015 Release (Version 2015.00) [Data set]. Palisades, NY: NASA Socioeconomic Data and Applications Center (SEDAC).<https://doi.org/10.7927/H4N014G5>. Accessed 2024-15-10. | 30 arc seconds (~1 km) | 2013 |
| Human Footprint Index | HFI | Mu, Haowei; Li, Xuecao; Wen, Yanan; Huang, Jianxi; Du, Peijun; Su, Wei; et al. (2021). An annual global terrestrial Human Footprint dataset from 2000 to 2018. figshare. Figure.<https://doi.org/10.6084/m9.figshare.16571064.v7>. Accessed 2024-15-10. | 1 km (30 arc seconds) | 2000:2018 |
|  |  | Hijmans RJ, Barbosa M, Ghosh A, Mandel A (2024). geodata: Download Geographic Data. R package version 0.6-2, [https://CRAN.R-project.org/package=geodata](https://cran.r-project.org/package=geodata) | 1 km (30 arc seconds) | 1993 |
| Human Population Density | HPD | Center For International Earth Science Information Network-CIESIN-Columbia University. (2017). Gridded Population of the World, Version 4 (GPWv4): Population Density, Revision 11 (Version 4.11) [Data set]. Palisades, NY: Socioeconomic Data and Applications Center (SEDAC). https://doi.org/10.7927/H49C6VHW. Accessed 2024-15-10 from<https://sedac.ciesin.columbia.edu/data/set/gpw-v4-population-density-rev11> | 2.5 arc minutes (~4x4 km) | 2000, 2005, 2010, 2015, 2020 |
| Road Density | RD | Meijer, J.R., Huijbregts, M.A.J., Schotten, C.G.J. and Schipper, A.M. (2018): Global patterns of current and future road infrastructure. Environmental Research Letters, 13-064006. Data is available at www.globio.info Accessed 2024-15-10. | 5 arc minutes (~8x8 km) | 2015 |
| Croplands | CR | Ramankutty, N., A.T. Evan, C. Monfreda, and J.A. Foley (2008), Farming the planet: 1. Geographic distribution of global agricultural lands in the year 2000. Global Biogeochemical Cycles 22, GB1003, doi:10.1029/2007GB002952. Accessed 2024-15-10 from<http://www.earthstat.org/cropland-pasture-area-2000/> | 5 arc minutes (~10 km) | 2000 |
| Pastures | PS | Ramankutty, N., A.T. Evan, C. Monfreda, and J.A. Foley (2008), Farming the planet: 1. Geographic distribution of global agricultural lands in the year 2000. Global Biogeochemical Cycles 22, GB1003, doi:10.1029/2007GB002952. Accessed 2024-15-10 from<http://www.earthstat.org/cropland-pasture-area-2000/> | 5 arc minutes (~10 km) | 2000 |

**Table S3**

Table S3: Median dispersal (km) as buffer radius and buffer size (km2) for each species.

| **Species** | **Median dispersal (km) as buffer radius** | **Buffer area (km²)** |
| --- | --- | --- |
| *Acinonyx jubatus* | 141.121872 | 62566.0203 |
| *Caracal caracal* | 98.5373597 | 30503.6434 |
| *Catopuma temminckii* | 54.8039395 | 9435.68488 |
| *Felis lybica* | 15.5236721 | 757.074805 |
| *Felis margarita* | 101.666039 | 32471.4495 |
| *Felis nigripes* | 31.036979 | 3026.27755 |
| *Felis silvestris* | 41.9763428 | 5535.52821 |
| *Herpailurus yagouaroundi* | 32.7854221 | 3376.84737 |
| *Leopardus geoffroyi* | 13.9626231 | 612.468703 |
| *Leopardus guigna* | 18.9298896 | 1125.76057 |
| *Leopardus guttulus* | 23.1767664 | 1687.54576 |
| *Leopardus pardalis* | 33.6458536 | 3556.41943 |
| *Leopardus tigrinus* | 15.5425223 | 758.914537 |
| *Leopardus wiedii* | 21.6711774 | 1475.41735 |
| *Leptailurus serval* | 44.4775778 | 6214.87115 |
| *Lynx canadensis* | 59.8399498 | 11249.4765 |
| *Lynx lynx* | 131.448264 | 54282.4674 |
| *Lynx pardinus* | 29.5911973 | 2750.90092 |
| *Lynx rufus* | 41.7196002 | 5468.02069 |
| *Neofelis nebulosa* | 44.6032588 | 6250.04368 |
| *Otocolobus manul* | 98.6377476 | 30565.8281 |
| *Panthera leo* | 196.173297 | 120900.934 |
| *Panthera onca* | 130.363991 | 53390.6447 |
| *Panthera pardus* | 110.555957 | 38398.492 |
| *Panthera tigris* | 113.884724 | 40745.6094 |
| *Panthera uncia* | 133.70741 | 56164.3611 |
| *Pardofelis marmorata* | 20.9072407 | 1373.23008 |
| *Prionailurus bengalensis* | 15.2630272 | 731.865425 |
| *Puma concolor* | 115.008535 | 41553.7304 |

**Table S4**

*Table S4: Phylogenetic signal for home range size assessed using Pagel’s λ method.*

| **Pagel’s λ** | **logL(λ)** | **Likelihood Ratio (λ = 0)** | **P-value** | **Phylogenetic Signal Detected** |
| --- | --- | --- | --- | --- |
| 0.176 | -176.95 | 0.641 | 0.423 | No |

**Figure S2**


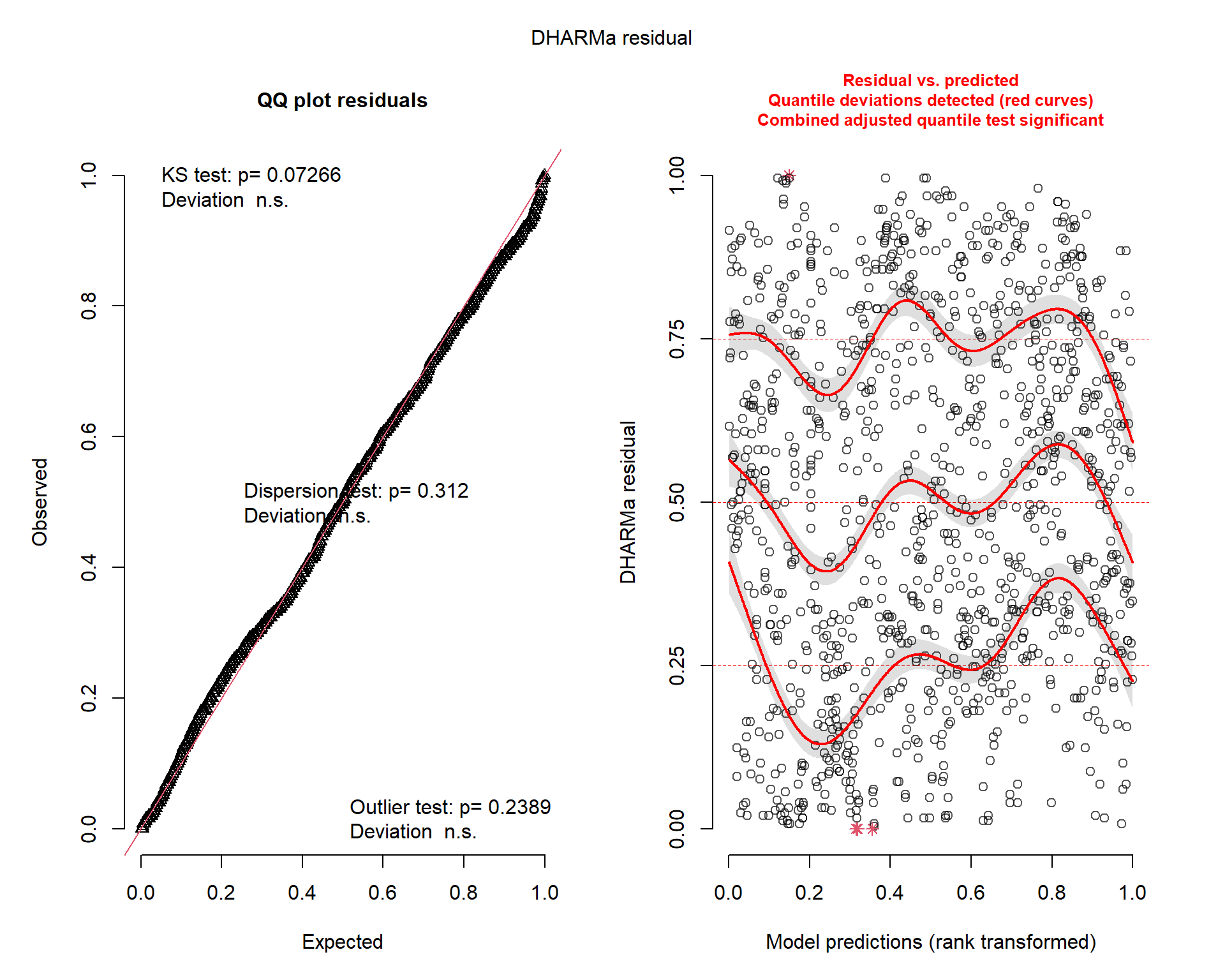


Figure S2: Residual diagnostics plot made with DHARMa for final HR model.

**Table S5**

*Table S5: Spatial autocorrelation for home range size assessed using Moran’s I test.*

| **Observed Moran’s I** | **Expected Moran’s I** | | **Standard Deviation** | **P-value** | **Spatial Autocorrelation Detected** |
| --- | --- | --- | --- | --- | --- |
| 0.0429 | | -0.0063 | 0.0509 | 0.3333 | No |

#### Supplement S5: Sensitivity analysis of the home range model

To assess the robustness and the contribution of each species to the pattern we have found, we conducted a leave‐one‐out (LOO) sensitivity analysis on the best‐supported GLMM for HR size. This model included ID nested within Species and Study ID as random intercepts, as well as intrinsic (Body mass and Sex), ecological (Net Primary Productivity and Felid Richness), anthropogenic (Croplands and Pastures), and methodological predictors (HR Estimation Method, Tracking Method and Number of Locations). It also incorporated interactions between Body mass and the anthropogenic predictors, as well as species-specific random slopes for all environmental predictors and Number of Locations. We sequentially removed all records of one species at a time and re-fitted this model structure on this reduced dataset, resulting in 29 models in total for 29 species, each estimating 12 parameters (11β coefficients plus the intercept).

For each LOO model, we compared its parameter estimates with those from the full model including all 29 species in three steps. First, we evaluated whether the significance category of each parameter matched that of the full model (<0.05, <0.10, or >0.10). Second, for parameters that remained significant, we checked whether the sign of the parameter matched that of the full model. Third, for parameters that remained significant and with concordant signs, we assessed whether the 95% confidence interval (CI) overlapped with that of the full model.

Based on these comparisons, each predictor in a LOO model was classified relative to its counterpart in the full model. A predictor was considered “robust” if all the three above mentioned criteria were met, “almost” if it experienced a slight shift in significance (i.e. parameter’s p-value changing from <0.05 to <0.10 or vice versa, without changes in sign or CI overlap) or “different” if it showed a larger deviation, such as a change in significance category, a sign mismatch, or non-overlapping confidence intervals. Classifications were determined independently for each predictor before summarizing the overall effect of species removal on the model’s biological interpretation.

If all twelve predictors (including the intercept) were classified as “robust” when removing one species, the model was considered “supported”, if only one predictor was classified as “almost,” the model was considered “partly supported”, and finally, if more than one predictor was classified as “almost” or at least one predictor was “different,” the model was considered as “not supported”.

Out of 29 LOO models, 5 did not converge (Table S6), notably convergence issues were not linked to the removal of the most‐represented species nor to those species with the largest or smallest median HR size (Figure S3). Of the 24 converged models, 83.3% resulted in the same biological interpretation as the full model (i.e. “supported”), increasing to 91% when “partly supported” models were also included. Four models deviated from the full model: two models showed slight changes in predictor significance without affecting their biological interpretation, while two models exhibited larger changes, with some interaction terms becoming significant when some species were removed (SupportingInformation_SensitivityAnalysis.xlsx). As such, when removing *Panthera pardus* or *Leopardus geoffroyi* the *p*‐value of pastures shifted slightly from <0.05 to <0.10. Moreover, when removing *Panthera tigris* and *Otocolobus manul,* the interactions between croplands and body mass, and between pastures and body mass, became significant, respectively. When parameters remained significant, the sign of the estimate always matched that of the full model, and CIs overlapped (see SupportingInformation_SensitivityAnalysis.xlsx).

Overall, our sensitivity analysis demonstrated the robustness of our model to describe home range variation when removing individual species (i.e. the pattern is supported regardless of the species removed, including the most represented and those showing extreme values for HR).

**Table S6:**

Table S6: Summary of the leave-one-out sensitivity analysis. For each species, the table reports whether the model converged, the number of records removed, the number of predictors classified as “Robust,” “Almost,” or “Different” out of 12 parameters (intercept included) and the resulting model sensitivity classification (“supported”, “partly supported”, or “not supported”). “Robust” predictors retained their significance, sign, and confidence interval overlap with the full model; “almost” predictors experienced minor shifts in significance without affecting their sign or CI overlap; “different” predictors showed larger deviations, such as changes in significance, sign, or non-overlapping CI. A model was considered “supported” if all predictors were “robust”, “partly supported” if only one predictor was “almost”, and “not supported” if more than one predictor was “almost” or at least one was “different”.

| Species removed | Converged | | | Records removed | “Robust” predictors | “Almost” predictors | “Different” predictors | Species  sensitivity  summary |
| --- | --- | --- | --- | --- | --- | --- | --- | --- |
| *Felis lybica* | | FALSE | 40 | |  |  |  |  |
| *Leopardus guigna* | | FALSE | 6 | |  |  |  |  |
| *Leopardus pardalis* | | FALSE | 42 | |  |  |  |  |
| *Leopardus tigrinus* | | FALSE | 1 | |  |  |  |  |
| *Panthera onca* | | FALSE | 83 | |  |  |  |  |
| *Acinonyx jubatus* | | TRUE | 15 | | 12 | 0 | 0 | Supported |
| *Caracal caracal* | | TRUE | 22 | | 12 | 0 | 0 | Supported |
| *Catopuma temminckii* | | TRUE | 6 | | 12 | 0 | 0 | Supported |
| *Felis margarita* | | TRUE | 12 | | 12 | 0 | 0 | Supported |
| *Felis nigripes* | | TRUE | 29 | | 12 | 0 | 0 | Supported |
| *Felis silvestris* | | TRUE | 11 | | 12 | 0 | 0 | Supported |
| *Herpailurus yagouaroundi* | | TRUE | 9 | | 12 | 0 | 0 | Supported |
| *Leopardus geoffroyi* | | TRUE | 36 | | 11 | 1 | 0 | Partly supported |
| *Leopardus guttulus* | | TRUE | 4 | | 12 | 0 | 0 | Supported |
| *Leopardus wiedii* | | TRUE | 7 | | 12 | 0 | 0 | Supported |
| *Leptailurus serval* | | TRUE | 45 | | 12 | 0 | 0 | Supported |
| *Lynx canadensis* | | TRUE | 28 | | 12 | 0 | 0 | Supported |
| *Lynx lynx* | | TRUE | 142 | | 12 | 0 | 0 | Supported |
| *Lynx pardinus* | | TRUE | 10 | | 12 | 0 | 0 | Supported |
| *Lynx rufus* | | TRUE | 156 | | 12 | 0 | 0 | Supported |
| *Neofelis nebulosa* | | TRUE | 6 | | 12 | 0 | 0 | Supported |
| *Otocolobus manul* | | TRUE | 4 | | 11 | 0 | 1 | Not supported |
| *Panthera leo* | | TRUE | 36 | | 12 | 0 | 0 | Supported |
| *Panthera pardus* | | TRUE | 80 | | 11 | 1 | 0 | Partly supported |
| *Panthera tigris* | | TRUE | 145 | | 11 | 0 | 1 | Not supported |
| *Panthera uncia* | | TRUE | 30 | | 12 | 0 | 0 | Supported |
| *Pardofelis marmorata* | | TRUE | 3 | | 12 | 0 | 0 | Supported |
| *Prionailurus bengalensis* | | TRUE | 6 | | 12 | 0 | 0 | Supported |
| *Puma concolor* | | TRUE | 123 | | 12 | 0 | 0 | Supported |

**Figure** **S3**


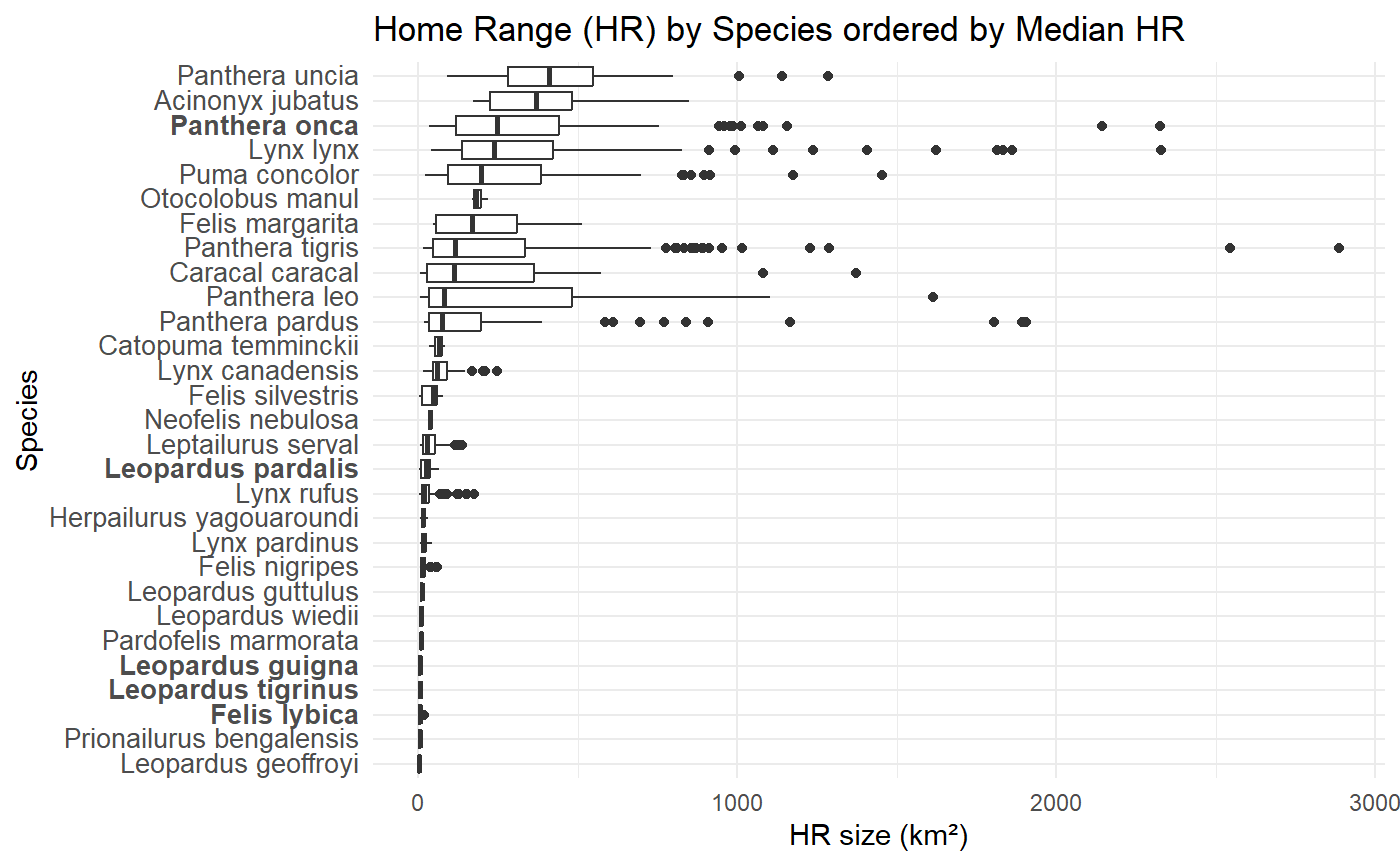


Figure S3: Home range size (km²) by species ordered by median Home range size. For species in bold, models did not converge.

**Table** **S7**

Table S7: Number of records by species present in Home Range (HR) dataset used for modelling. The total number of records is presented in bold.

| **Species** | **Number of records in HR dataset** |
| --- | --- |
| *Acinonyx jubatus* | 15 |
| *Caracal caracal* | 22 |
| *Catopuma temminckii* | 6 |
| *Felis lybica* | 40 |
| *Felis margarita* | 12 |
| *Felis nigripes* | 29 |
| *Felis silvestris* | 11 |
| *Herpailurus yagouaroundi* | 9 |
| *Leopardus geoffroyi* | 36 |
| *Leopardus guigna* | 6 |
| *Leopardus guttulus* | 4 |
| *Leopardus pardalis* | 42 |
| *Leopardus tigrinus* | 1 |
| *Leopardus wiedii* | 7 |
| *Leptailurus serval* | 45 |
| *Lynx canadensis* | 28 |
| *Lynx lynx* | 142 |
| *Lynx pardinus* | 10 |
| *Lynx rufus* | 156 |
| *Neofelis nebulosa* | 6 |
| *Otocolobus manul* | 4 |
| *Panthera leo* | 36 |
| *Panthera onca* | 83 |
| *Panthera pardus* | 80 |
| *Panthera tigris* | 145 |
| *Panthera uncia* | 30 |
| *Pardofelis marmorata* | 3 |
| *Prionailurus bengalensis* | 6 |
| *Puma concolor* | 123 |
| **Total** | **1137** |

**Figure S4**


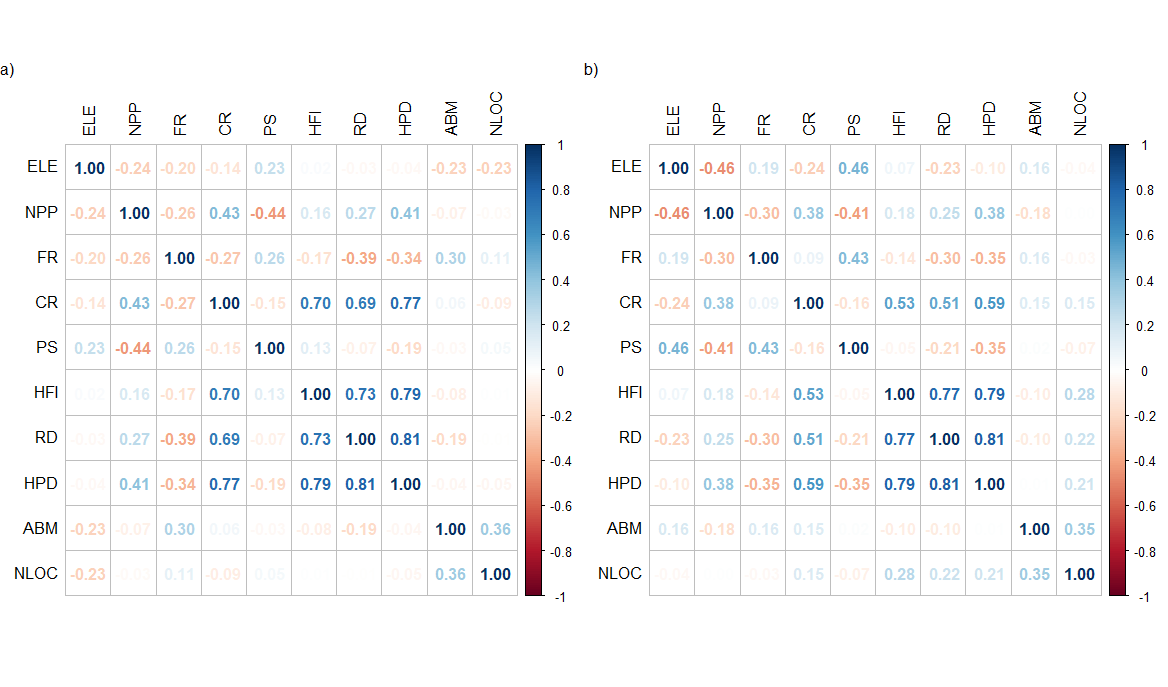


Figure S4: Correlation plot for HR dataset. Human population density (HPD) and Human Footprint Index (HFI) are highly correlated with Croplands (CR) and Road Density (RD). They are therefore excluded from the analysis.

**Table S8**

*Table S8: Anova table comparing random effect structures in initial GLMM model for HR dataset a) with or without Genus and b) with or without Study ID.*

*(a)*

| **Model** | **Random Structure** | **Df** | **AIC** | **logLik** | **Deviance** | | **Chisq** | **Chi Df** | **Pr (>Chisq)** |
| --- | --- | --- | --- | --- | --- | --- | --- | --- | --- |
| *ABM + SEX + HRM + TM + NLOC + ELE + NPP + FR + RD + RD:ABM + CR + CR:ABM + PS + PS:ABM* | *(1\| Species/ID) + (1\|Study ID)* | 19 | 11538.3 | -5750.149 | | 11500.3 | NA | NA | NA |
| *ABM + SEX + HRM + TM + NLOC + ELE + NPP + FR + RD + RD:ABM + CR + CR:ABM + PS + PS:ABM* | *(1\| Genus/ Species/ID) + (1\|Study ID)* | 20 | 11540.3 | -5750.149 | | 11500.3 | 0 | 1 | 1 |

(b)

| **Model** | **Random Structure** | **Df** | **AIC** | **logLik** | **Deviance** | | **Chisq** | **Chi Df** | **Pr (>Chisq)** |
| --- | --- | --- | --- | --- | --- | --- | --- | --- | --- |
| *ABM + SEX + HRM + TM + NLOC + ELE + NPP + FR + RD + RD:ABM + CR + CR:ABM + PS + PS:ABM* | *(1\| Species/ID)* | 18 | 11704.19 | -5834.10 | | 11668.19 | NA | NA | NA |
| *ABM + SEX + HRM + TM + NLOC + ELE + NPP + FR + RD + RD:ABM + CR + CR:ABM + PS + PS:ABM* | *(1\| Species/ID) + (1\|Study ID)* | 19 | 11538.30 | -5750.15 | | 11500.30 | 164.90 | 1 | 0 |

**Table S9**

Table S9: Parameter estimates for home range size prediction after model averaging (conditional average reported).

| Parameter | Estimate | SE | Adjusted SE | Z value | P-value | |  |
| --- | --- | --- | --- | --- | --- | --- | --- |
| INTERCEPT | 3.81117 | 0.20884 | 0.20907 | 18.229 | | < 2e-16 | *** |
| CR | -0.52903 | 0.08687 | 0.08696 | 6.083 | | < 2e-16 | *** |
| ELE | 0.22116 | 0.08764 | 0.08773 | 2.521 | | 0.011705 | * |
| FR | -0.33533 | 0.08877 | 0.08887 | 3.773 | | 0.000161 | *** |
| HRM (MCP) | 0.18715 | 0.03279 | 0.03282 | 5.702 | | < 2e-16 | *** |
| ABM | 0.90679 | 0.15323 | 0.15340 | 5.911 | | < 2e-16 | *** |
| NLOC | 0.19752 | 0.03294 | 0.03298 | 5.990 | | < 2e-16 | *** |
| NPP | -0.28306 | 0.06521 | 0.06528 | 4.336 | | 1.45e-05 | *** |
| PS | -0.11872 | 0.06884 | 0.06891 | 1.723 | | 0.084945 | . |
| SEX (M) | 0.51911 | 0.06635 | 0.06642 | 7.816 | | < 2e-16 | *** |
| CR:ABM | 0.16386 | 0.07391 | 0.07398 | 2.215 | | 0.026775 | * |
| PS:ABM | 0.13357 | 0.06223 | 0.06230 | 2.144 | | 0.032032 | * |
| TM (SAT) | 0.07540 | 0.16953 | 0.16972 | 0.444 | | 0.656831 |  |
|  |  |  |  |  | |  |  |

**Table S10**

*Table S10: Variance components of random effects in the final GLMM for HR size.
Random effects include individual nested within species (ID.Species), species intercepts, study ID, and species-level slopes for environmental and anthropogenic predictors.*

| **Random effect** | **Term** | **Variance** | **Std. Dev.** |
| --- | --- | --- | --- |
| ID.Species | (Intercept) | 0.197 | 0.444 |
| Species | (Intercept) | 0.745 | 0.863 |
| Study_ID | (Intercept) | 0.312 | 0.559 |
| Species slope | ele | 0.138 | 0.371 |
| Species slope | npp | 0.000 | 0.000005 |
| Species slope | cr | 0.163 | 0.404 |
| Species slope | ps | 0.000 | 0.000001 |
| Species slope | log_nloc | 0.007 | 0.082 |
| Species slope | cr:log_abm_sex | 0.000 | 0.00018 |
| Species slope | ps:log_abm_sex | 0.000 | 0.000002 |
| Species slope | fr | 0.000 | 0.0007 |
